# *In vivo *myograph measurement of muscle contraction at optimal length

**DOI:** 10.1186/1475-925X-6-1

**Published:** 2007-01-02

**Authors:** Niels Rahe-Meyer, Christian Weilbach, Matthias Karst, Matthias Pawlak, Aminul Ahmed, Siegfried Piepenbrock, Michael Winterhalter

**Affiliations:** 1 Department of Anaesthesiology, Hannover Medical School, Carl-Neuberg-Str. 1, D-30625 Hannover, Germany; 2 Department of Anaethesiology, St. Josefs Stift Cloppenburg, Krankenhausstr. 13, D-49661 Cloppenburg, Germany; 3 Institute of Physiology, University of Wuerzburg, Roentgenring 9, D-97070 Wuerzburg, Germany; 4 St. Thomas' Hospital, Lambeth Palace Road, London SE1 7EH, Great Britain

## Abstract

**Background:**

Current devices for measuring muscle contraction *in vivo *have limited accuracy in establishing and re-establishing the optimum muscle length. They are variable in the reproducibility to determine the muscle contraction at this length, and often do not maintain precise conditions during the examination. Consequently, for clinical testing only semi-quantitative methods have been used.

**Methods:**

We present a newly developed myograph, an accurate measuring device for muscle contraction, consisting of three elements. Firstly, an element for adjusting the axle of the device and the physiological axis of muscle contraction; secondly, an element to accurately position and reposition the extremity of the muscle; and thirdly, an element for the progressive pre-stretching and isometric locking of the target muscle.

Thus it is possible to examine individual *in vivo *muscles in every pre-stretched, specified position, to maintain constant muscle-length conditions, and to accurately re-establish the conditions of the measurement process at later sessions.

**Results:**

In a sequence of experiments the force of contraction of the muscle at differing stretching lengths were recorded and the forces determined. The optimum muscle length for maximal force of contraction was established. In a following sequence of experiments with smaller graduations around this optimal stretching length an increasingly accurate optimum muscle length for maximal force of contraction was determined. This optimum length was also accurately re-established at later sessions.

**Conclusion:**

We have introduced a new technical solution for valid, reproducible *in vivo *force measurements on every possible point of the stretching curve. Thus it should be possible to study the muscle contraction *in vivo *to the same level of accuracy as is achieved in tests with *in vitro *organ preparations.

## Background

The *in vivo *diagnostic examination of life threatening illnesses affecting the musculoskeletal system is of clinical significance. Muscle function has been extensively studied *in vitro *but there are some important differences to the *in vivo *setting. For example, *in vivo *the effect of surrounding muscular and non muscular structures lead to inhomogeneous sarcomer lengths. That means that even at the optimum muscle length in a setting not in all sarcomers all myosin heads can reach a connection point on its accompanying actin filament. [1,2] In addition, amplitude and length of the *in vitro *and the *in vivo *contraction are not equal because the serial elastic elements of the *in vivo *muscles (for instance the tendons) diminish the amplitude and prolong the duration of contraction.

### The present technology for *in vivo *examination of muscle contraction

Many devices used in the *in vivo *examination of muscle contraction exist. Some are used in the clinical setting (such as in muscle relaxometry), while others are used solely for experimental studies. However, none are used for routine clinical examination of diseases of the motor system, for this the gold standard is the electromyography [3-5].

One category of devices examines the maximum voluntary isometric contraction. This includes the force of the handgrip in the clinical setting [6] and the contraction force of muscle groups under laboratory conditions [7-13]. Maximum voluntary contractions are difficult to define in relation to the muscles involved, and are subject to increased variability compared to electrically stimulated contractions. All the known devices for voluntary contractions have no pre-stretching mechanism and have an imprecise setting mechanism, therefore the position of the extremity and the length of the muscle is determined by the conditions of the measuring equipment. By this a good reproducibility might be achieved [6,10,14] but the results cannot lead to absolute, valid statements of the examined muscle independent from the measuring conditions.

Another category of devices use electric stimulations as a contraction trigger and thus produce standardized muscle contractions. The question remains whether they can process these contractions accurately. In none of the known devices it is described how an extremity can be set so that the muscle contraction transmits fully to the contraction sensor independent of the different morphology of the test subject and independent of the stretching of the muscle.

### Technical aims of an ideal measuring device and goals of the study

An ideal measuring device for *in vivo *testing should have the following characteristics:

i. The device should include an accurate and reproducible element for adjusting the physiological axle and for setting the extremity. The muscle contraction should fully transmit to the measuring device independent of the experimental conditions.

ii. The device should include an element to stretch the muscle and fix it into position. The stretching-element should allow accurate muscle examination at its optimum length. For later sessions the former optimum length should be reproducible, thus allowing only minor corrections to adapt to the specific *in vivo *conditions.

iii. The measuring conditions should not alter or restrict the results so that objectivity, reliability and validity of the *in vivo *examination are to the same accuracy as in *in vitro *tests.

This study should provide evidence for a measuring device that records an exact stretching curve with a related force contraction curve and a defined optimum muscle length. The extremity setting of the device should be flexible enough to deal with test subjects with differing extremity sizes and should hold a constant vector during all possible stretching lengths. The connected muscle length together with the setting data should be storable and reproducible for later sessions with the same test subject.

## Methods

This section describes the most important structural aspects of a novel device for accurate and reliable measurement of muscle force and describes the method with which the experiment for measuring the optimum stretching length and the individual muscle force was performed.

### Axle-adjustment design

**Figure 1:**
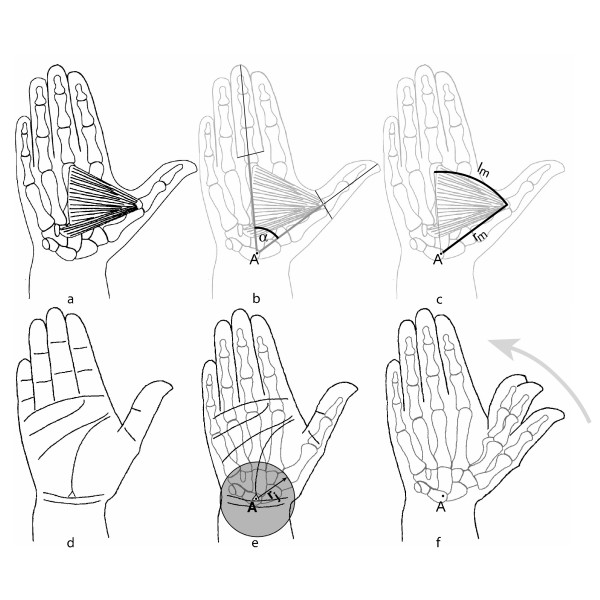
**a-f: Anatomical relations of the *musculus adductor pollicis***. The fan shaped *musculus adductor pollicis *stretches in the hand, and originates mainly from the span of the third metacarpal the finger, inserting into the metacarpal joint of the index finger (**Fig. 1a, f**). The muscle acts around a rotating joint which forms a circular arc around an imaginary pivot A, which lies at the distance r_j _outside the joint area (**Fig. 1e**). With the radius r_m _of this pivot A, the ideal muscle length (l_m_) lies as an arc between the base and the origin (**Fig. 1c**). The muscle length l_m _can be determined through measuring the muscle angle α and the muscle radius r_m_. (**Fig. 1b, c**). .

The tests were performed on the *musculus adductor pollicis*. The muscle is structured like a fan and can be approximated to an arc of a circle (see l_m _in Fig. 1c), an implicit convention for 30 years [15]. The physiological rotation axis runs outside the cylindrical joint and remains constant at all muscle lengths (see Fig. 1e–f).

**Figure 2:**
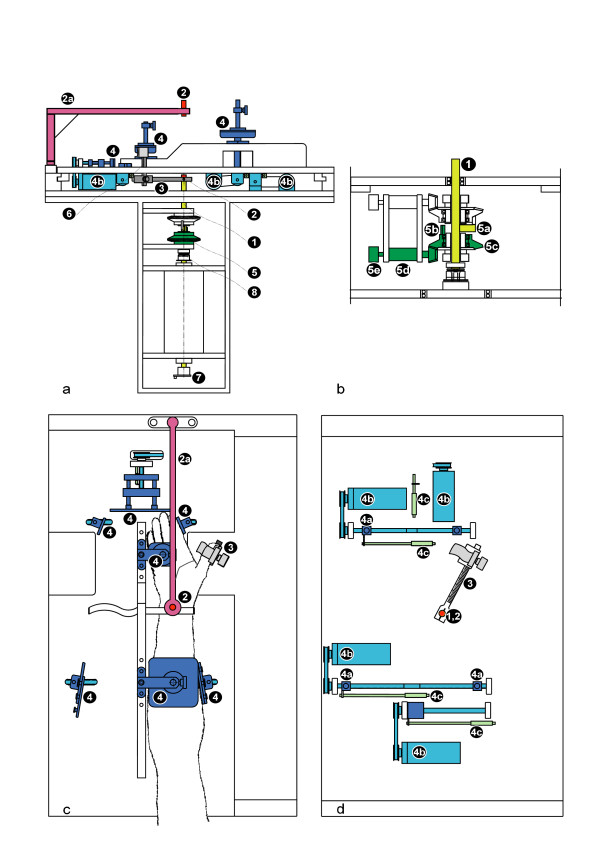
**a-d: Constructive elements**. **Fig. 2a **shows the apparatus in side view, having removed the front cover plate; **Fig. 2b **shows a detailed sectional view of the infinitely variable adjustable stopping mechanism; **Fig. 2c **shows the top view onto the extremity-setting of the apparatus as does **Fig. 2d **after removal of the arm rest plate. List of technical components (colors in brackets): (1) = axle (yellow) (2) = laser beamer (red) (2a) = swivel arm (lavender) (3) = thumb lever (dark gray) (4) = supporting cushion (blue) (4a) = supporting cushion mount (blue) (4b) = adjustment motors (light blue) (4c) = setting measuring gauge (green blue) (5) = stopping mechanism unit (5a) = bolt on axle (yellow) (5b) = isometric stopping bolt (green) (5c) = adjustment wheel (green) (5d) = adjustment motor (green) (5e) = stopping mechanism gauge (green) (6) = beam-measuring gauge (gray) (7) = potentiometer (8) = adjustable friction clutch. **Fig. 2a,c,d **show the adjusting and extremity-setting design. The thumb can be set at the thumb lever **(3)**, which is fixed to the main axle **(1) **around which it can rotate. The setting elements **(4) **are for the setting of the hand and arm. The supporting cushions lead to the extremities and lock in place via mounting blocks **(4a)**. Electro-motors hold the arm into place **(4b) **and the muscle position is determined via the splint-measuring gauge **(4c)**. At the end of both the apparatus axle **(1) **and the end of the swivel arm **(2a)**, there are lasers **(2)**, whose beams elongate the apparatus axle and project onto the extremity (hand) to be inserted. **Fig. 2c **shows the right arm and hand set in position. The cushions are symmetrically assembled for adjustment and setting of the left arm and hand. To achieve this, the arm rest plate is moved to the right, ensuring the left arm is over the axle. **Fig. 2a-b **show the pre-stretching and isometric stopping mechanism which consists of an isometric bolt **(5b) **which can be variably adjusted by means of an adjustment wheel **(5c) **and adjustment motor **(5d)**. Thus the isometric bolt **(5b) **takes the stopping bolt of the axle **(5a) **with it and, therefore, indirectly rotates the axle **(1) **and the thumb lever **(3) **into the desired position. An adjustable friction clutch **(8) **prevents an overstretching of the muscle.

The aim is that the physiological rotation axis can be made congruent with the axle of the apparatus – so that the extremity is not shifting in the setting with another pre-stretching muscle lengths and the muscle forces entirely interact with the measuring device. To achieve this one laser beam runs directly through the axle of the apparatus (see 1, 2 in Fig. 2a, c), and a second beam is projected by a swivel arm and marks the same axle (see 2a, 2 in Fig. 2a, d). In this way, spots are projected on to the dorsal and ventral aspect of the hand, which is adjusted until the projected laser spots are congruent with the anatomical reference points of the hand and wrist for the physiological rotation axis (see A in Fig 1).

### Extremity-setting design

When the hand is in the correct position, mounted cushion pads (see 4a, 4 in Fig. 2) are moved towards the extremity (arm and hand) using spindles powered by electro-motors (see 4b in Fig. 2d) and set into position. The power of the electro-motors is limited so that even with full power no harm could be done to the test subjects. The precise position of these cushions is determined by electronic measurement recorders (see 4c in Fig. 2d), which save the positional information from the first session and can, therefore, automatically be moved into the exact position at later sessions [16-18]. The extremity setting design can take in right or left, pronated or supinated hands. If the examination of another muscle, for instance an adductor of the leg, is required the extremity setting module can be removed at the axle and another module specific for the setting of larger muscles can be mounted in its position. [16,17,19]

### Pre-stretching design

A pre-stretching mechanism has been developed, where the muscle can be exactly stretched to any physiological length and locked into place isometrically.

The pre-stretching mechanism is suspended to the axle of the apparatus (see 5 in Fig. 2a–b). An isometric stopping bolt (5b) can be adjusted infinitely variable by means of an adjustment wheel (5c) and an adjustment motor (5d). The isometric stopping bolt (5b) contacts the bolt of the axle (5a) and thereby rotates the axle (1), moving the thumb lever (3), and stretching the muscle. When the isometric stopping bolt is in the defined position it is automatically locked into place and the muscle length is isometrically fixed. In a pre-test the maximum stretching force for a test-subject is individually determined by stepwise increment until no relevant length stretching occurs or further stretching is uncomfortable for the testsubject. This is rare even with the maximum stretching force of 60 Newtons. To prevent an overstretching of the muscle an adjustable friction clutch (8) is installed in the axle. Each position of the stopping mechanism is determined with an electrical measurement recorder (5e). This information is stored and the mechanism can be moved automatically into position for the same subject at a later point in time. [16-18]

### Stimulation design

The muscle contractions are triggered indirectly via the *nervus ulnaris *which is activated at the wrist by surface electrodes. The nerve stimulator produces electric pulses of a rectangular form with durations between 0.01 ms and 1000 ms, pulse numbers between 1 and 99, amplitudes between 0.1 mA and 50 mA, frequencies between 1250 Hz and 0.01 Hz (although not all combinations are possible because of a limited power in the system). In the presented experiments the pulses have a duration of 0.1 ms with a frequency of one pulse every 100 seconds. The amplitude of the currency pulse was individually determined in a pre-test. This was 15% higher than necessary for a maximum amplitude of the mechanical peak twitch contraction and is thus called the supra-maximum pulse with which all motor units of the *musculus adductor pollicis *are simultaneously stimulated once with each pulse [16,18,19].

### Measurement methods

Forces of pre-stretching and of contraction are measured at the thumb lever with a beam-measuring gauge (see 6 in Fig. 2a). The muscle lengths are determined via a potentiometer (see 7 in Fig. 2a) on the apparatus axle (see 1 in Fig. 2a), which determines the angle of the thumb lever (see α in Fig. 1b). To convert the quantified data into muscle force and muscle length, the length of the muscle radius (see r_m _in Fig. 1c) and the length of the thumb lever (see 3 in Fig. 2a, d) from the middle of the axle to the middle of the beam-measuring gauge are determined (see 1, 6 in Fig. 2a). The idealized muscle length (see l_m _in Fig. 1c) is interpreted as an arc defined by the distance r_m _between the pivot A and the base of the muscle and by the angle α from the pivot A between the base of the muscle and the corresponding point of the third finger (see Fig. 1c).

### Experimental method to determine the optimum stretching length and the individual muscle force

The extremity of the test subject is adjusted and set in the device. The stimulation electrodes are placed on the skin over the *nervus ulnaris *at the level of the wrist. With sub-maximal test pulses the position of the electrodes are optimized. In a first pre-test the amplitude of the supra-maximal stimulus is determined with increasing electric pulses. In a second pre-test the maximum stretching-force is determined with incremental stretching forces.

The main test is performed in a sequence of 10 experiments with different stretching forces in each experiment. At the beginning of each experiment the muscle is stretched for approximately five seconds ensuring the process of stretching is finished. A supra-maximal stimulus leads to a force contraction after which the stretching force returns to zero. The first main-test sequence had stretching forces from zero to maximum. Stretching lengths and forces and the contraction forces are recorded, the maximum contraction is with the optimum stretching force at the optimum length.

In a second main-test sequence stretching forces from one before to one after the optimum of the first sequence are used resulting in finer steps around the optimum of the first sequence.

## Results: measuring example of rest-stretching and isometric maxima

**Figure 3:**
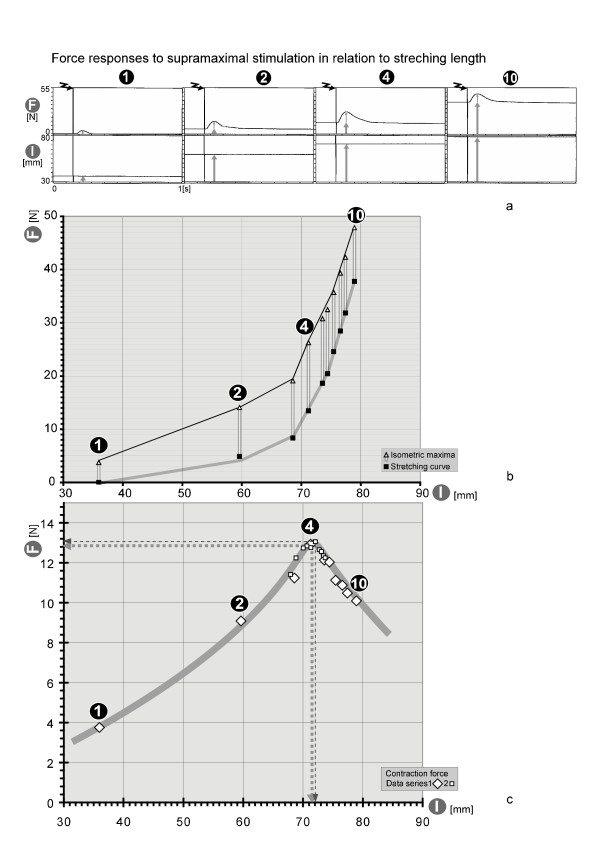
**a-b: Isometric maxima function + optimum pre-stretching function**. **Fig. 3a **shows four examples of the original recordings of force (F) and muscle length (l; taken from a sequence of ten). The amplitudes of the force of contraction responses (small arrows) to single supra maximum stimulation are determined with increasing pre-stretching forces or increasing muscle lengths. **Fig. 3b **shows the stretching forces (squares) and the contraction forces (arrows) in relation to the stretching lengths. In **Fig. 3c **the stretching forces are omitted and the contraction forces (big lozenges) are shown in relation to the stretching length. In 4 of the series, the optimum pre-stretching length is largely achieved with a muscle length of l = 71 mm. The small lozenges show the results of a second sequence of ten experiments with smaller graduations around the optimum stretching length which was determined by the first sequence (l = 71 mm), so that the accuracy of the optimum length can be further elucidated at a more precise length = 72.1 mm.

Fig. 3a shows four examples of a sequence of ten experiments (#1, 2, 4, 10). The top fields indicate no force in Part 1 of the experiment, whereas a stretching force of nearly 40 N was used in Part 10 (indicated by wide arrows). The bottom fields show the muscle lengths, which increase with the stretching force (indicated by wide arrows). With each stretching force or with each extension of the muscle, a single supra-maximum stimulus was fired for 0.2 ms (zigzag arrow), achieving a standardized contraction in which all muscle fibres were activated simultaneously. These contractions (thin arrows) can be seen as a force increase, with an optimum at around 0.25 ms in the higher fields. The contraction forces are small at low muscle-stretching lengths (Part 1), peak at longer muscle-stretching lengths (Part 2 and 4) and decrease at the longest muscle-stretching lengths (Part 10). In the graph representing the optimal contraction forces in relation to muscle lengths (Fig. 3b), the muscle's typical optimum stretching length (approximately 71 mm) can be determined, along with the optimum single-stimulus contraction force (approximately 13 N). In Fig. 3c the small lozenges show the results of a second sequence of experiments with smaller graduations around the previously determined optimum stretching length. In this way the optimum length of the muscle can be determined accurately: length = 72.1 mm.

## Discussion

A new measuring device to examine the force of the human *musculus adductor pollicis invivo *has been presented here. In contrast to the given literature we presented a clear definition how a muscle with a complicated form like the *musculus adductor pollicis *can be idealized so that lever forces and positions can be related to muscle forces and length *invivo*.

One major advantage compared to known devices is the mechanisms for a precise axle adjustment and automatic setting (see "Methods"). These allow an implementation on the different morphology of the test subjects and a complete and constant transmission of the muscle vector on the force sensor independently from the muscle stretching length. The axle adjustment and setting values are stored and can be exactly reproduced at later sessions.

Current literature details no description on how the pre-stretching axle and the contraction-force vector is held constant. Importantly, the known devices have no technical designs for axle-adjustment and extremity-setting.

i. Current devices do not define the axle of the device, nor determine the physiological axis. They are unable to bring the axle in congruent extension to the physiological axis [20-28]. Sometimes limitations of the devices do not allow the adaptation for the sizes of different test subjects, e.g. due to a fixed distance between the ground board and axle [13,29,30] and a simplified parallel relation between them [13].

ii. No device has a reproducible, constant extremity-setting. Indeed, one device fixes the extremity such that muscle contraction is impeded [31].

iii. With no precise axle-adjustment and constant extremity-setting the contraction-force vector in addition to the length of the connected levers and of the muscle are undefined and change if the muscle is stretched to different lengths.[21-27,29,30]

Another major advantage compared to known devices is the presented stretching mechanism. With a pre-stretching mechanism it should be possible to bring the muscle to an optimum length. Many known devices have no pre-stretching mechanism for the optimum length but use an arbitrary length [15,25,27,32] or define the length by the stretching force [20,22]. Other devices have a pre-stretching mechanism, but these are graded either with rough gradation [31] or with finer gradations using a step-motor [21,29,30].

A pre-stretching mechanism which sets the muscle length according to a particular value of the pre-stretch force is not suitable because the same stretching force will lead to different muscle lengths. A graded pre-stretching mechanism can be too inaccurate to examine the muscle at its optimum length – only with an infinitely variable pre-stretching mechanism the optimum length can be established.

The reinstatement of the extremities, the repeated changing of position during the examination, and the sub-optimal pre-stretching all lead to a reduction in accuracy of the measurement of contraction force. The measurement results gained in this way are at best semi-quantitative and only suitable to examine changes within a series of measurements. For instance, twitch monitors in anaesthesiology have no mechanism for axle-adjustment, extremity-setting or pre-stretching, but are sufficient to semi-quantitatively estimate the intra-operative effect of muscle relaxants. But the clinical applications of devices with only semi-quantitative measurement methods are limited.

The setting elements we presented in the myograph are built in modular technique so that they can simply be substituted for another setting module for larger dimensions. By this we examined several small muscles including the *abductor pollicis longus *and *brevis*, the *opponens pollicis*, and the *interossei palmares*, and some larger muscles including the *musculus brachialis*, the *biceps brachii*, the *triceps brachii*, the *tibialis anterior*, and the *musculus triceps surae*. The majority of our examinations, however, were conducted on the *musculus adductor pollicis *due to three main reasons:

i. The abduction of the thumb can be performed to over 40 degrees, stretching *musculus adductor pollicis *to 275%. Consequently, a large part of the stretching curve may be examined, including the optimum length. In contrast to this there are many structural problems with the examination of long muscles *in vivo*. Since their base and origin reach asymmetrically over the joint, a large movement in the joint corresponds only with little stretching of the muscle. Thus only a small section of the stretching curve – mostly without a clear optimum length – can be examined. Further more, the stretching of large muscles is often associated with a considerable change in the force-vector [33] even more when they are associated with smaller muscles [34].

ii. The *adductor pollicis *can be easily stimulated via the *ulnar nerve *at the level of the wrist. By this at the radial side only two muscles are activated: the *musculus adductor pollicis *and *the flexor pollicis brevis*. Both muscles can be seen as a functional association with the same origin, same insertion and same physiological axis for the thumb movement. Their contractions are completely transmitted to the force sensor. At the ulnar side the *ulnar nerve *activates some other muscles too, their contractions, however, are buffered by the setting cushions and their contractions do not contribute to the force output which is measured.

iii. The *musculus adductor pollicis *is a target muscle of electromyographical and mechanomyographical investigations and accordingly many devices concentrate on this muscle (see above) resulting in it being the most commonly investigated *in vivo *human muscle.

This means on the other hand that the examination of other muscles than the *musculus adductor pollicis *has its mentioned limitations for in-vivo examination independent from the type of measuring device. These limitations are of no clinical importance if one have a muscular disease in which all muscles are equally affected – then one best examines a small peripheral muscle like the *adductor pollicis *as a pars pro toto.

Before the experiment the intensity of the supra-maximal stimulus has to be defined either over the amplitude (or integral) of the EMG M-wave or the amplitude (or integral) of the acoustic response or the force amplitude of the mechanical twitch. All three parameters reach their maximum amplitude at approximately the same stimulation level [35-38]. The presentment myograph has the possibility to record all these parameters simultaneously, although is not described in the method section to keep it coherent. We use the force measurement for determination of the supra-maximal stimulus because it is a quantitative signal, insensible to electrical fields and not depending on the placement of the sensors.

The pre-stretching design of the myograph allows an infinitely variable stretching of the muscle. For an exact determination of the optimum length a single main-test sequence over the whole stretching curve is approximate, so that a second main test sequence with finer stretching progressions around the optimum of the first sequence is performed. For later sessions the optimum muscle length of the previous testing is stored. Although the previous optimum can differ slightly from the actual optimum due to myofascial force transmission, the previous optimum is situated very near the actual optimum.[1,2] Thus not the whole stretching curve must be examined a new but only a little part around the previous optimum in fine graduation to determine the actual optimum length accurately (see small lozenges Fig. 3c).

This results in an automized handling of the measuring device and also a greater reliability compared with all other known devices.

The myograph is used in several universities in Germany for the education of students of medicine and biology. Instead of depending on preparations of frog organs to learn how nerve and muscles work they now can experiment on themselves.

In the clinical context we use the myograph to ascertain the individual strength and we examine the changes in the strength over a period of time in repeated tests. We use the device on our intensive care unit for ventilated patient with weaning failure to decide if the strength of the patient is good enough for an attempted extubation. Additionally we use it prognostically in the monitoring of the development of a patient's polyneuropathy/myopathy illness on the intensive care unit.

The example in Fig. 3a–b shows that with presented apparatus, individual optimum muscle length can be accurately measured. So that the presented device indeed is comparable in the experimental conditions and results with well established *in vitro *tests [39].

The performance capacity of the individual muscle can be quantified through the contraction force at optimum stretching length. No changes in length are made during the isometric contractions (Fig. 3a). The examinations are therefore comparable to *in vitro *experiments on whole muscle preparations. However, with both experimental methods there are muscle length fluctuations during the contraction which cannot be measured. These fluctuations are a result from the torsions of the measuring apparatus (axle and thumb lever) and through stretching of elastic elements within the muscle itself (e.g. the tendons) at the expense of contractile elements.

## Conclusion

The axle of the apparatus and the physiological axis can be made congruent reliably: the extremity is set in this position and during the taking of these measurements, the anatomical conditions can be kept constant and automatically be restored for later sessions. An infinitely adjustable pre-stretching element makes examinations with optimum pre-stretching possible and this individual muscle length is likewise measured and stored for later sessions. With these, the technical solutions are being introduced as a prerequisite for valid, reproducible *in vivo *force measurements.

This myograph is already used for physiology teaching in many universities in Germany and thereby replaces *in vitro *frog experiments. In addition clinical studies with a prototype of this myograph have been started in the field of critical illness neuro-myopathy which show quantitative, repeatable, valid and accurate reports about the individual efficiency of the muscles of a patient and it changes through time. Additionally with the device (and simultaneously recorded electromyogram) we can differentiate between the affection of nerve or muscle in this disease – and adapt the therapy accordingly. [40,41] This has yielded new perspectives for the diagnosis and therapy control of muscle illnesses.

## Authors' contributions

NRM developed the device and wrote the manuscript, NRM, MW, CW, and MK carried out the experiments on the different generations of laboratory devices on its way to a serial product, MW and SP co-ordinated the testing. MP helped with the physiological interpretation of the data, MW, SP, and AA helped to draft the manuscript.
